# Action of Platelet-Rich Plasma on In Vitro Cellular Bioactivity: More than Platelets

**DOI:** 10.3390/ijms24065367

**Published:** 2023-03-10

**Authors:** Maider Beitia, Diego Delgado, Jon Mercader, Pello Sánchez, Leonor López de Dicastillo, Mikel Sánchez

**Affiliations:** 1 Advanced Biological Therapy Unit, Hospital Vithas Vitoria, 01008 Vitoria-Gasteiz, Spain; 2 Microfluidics Cluster UPV/EHU, BIOMICs Microfluidics Group, Lascaray Research Center, University of the Basque Country UPV/EHU, 01006 Vitoria-Gasteiz, Spain; 3 Arthroscopic Surgery Unit, Hospital Vithas Vitoria, 01008 Vitoria-Gasteiz, Spain

**Keywords:** platelet-rich plasma, growth factors, platelets, insulin-like growth factor-1, IGF-1, cell proliferation

## Abstract

Platelet-rich plasma (PRP) is a biological therapy in which one of the mechanisms of action is the stimulation of biological processes such as cell proliferation. The size of PRP’s effect depends on multiple factors, one of the most important being the composition of PRP. The aim of this study was to analyze the relationship between cell proliferation and the levels of certain growth factors (IGF-1, HGF, PDGF, TGF-β and VEG) in PRP. First, the composition and effect on cell proliferation of PRP versus platelet-poor plasma (PPP) were compared. Subsequently, the correlation between each growth factor of PRP and cell proliferation was evaluated. Cell proliferation was higher in cells incubated with lysates derived from PRP compared to those cultured with lysates derived from PPP. In terms of composition, the levels of PDGF, TGF-β, and VEGF were significantly higher in PRP. When analyzing the PRP growth factors, IGF-1 was the only factor that correlated significantly with cell proliferation. Of those analyzed, the level of IGF-1 was the only one that did not correlate with platelet levels. The magnitude of PRP’s effect depends not only on platelet count but also on other platelet-independent molecules.

## 1. Introduction

Platelet-rich plasma (PRP) is a biologic therapy within the framework of regenerative medicine, the intention of which is to exploit the therapeutic potential of platelets. For this purpose, a small volume of the patient’s blood is collected to obtain a plasma fraction with a concentration of platelets similar to or higher than blood levels. The exogenous or endogenous activation of platelets present in this plasma fraction leads to the release of their biomolecular content, which will act on target tissues and cells [1]. The biosafety of PRP, thanks to its autologous nature as well as its ease of collection and application, have made it a widely used treatment in numerous medical fields [2,3,4,5].

Despite promising results and the increasing volume of research on PRP, there is still a need to better understand the mechanisms of action through which it carries out its work. However, it seems to be well established that many of the actions of PRP occur through the modulation of cellular activity [6]. Thus, numerous studies have shown that PRP stimulates cell proliferation, which is a key process in the treatment of pathologies that require cellular restoration of tissue, such as degenerative lesions and wound-healing processes. This has been observed in different cell populations, such as chondrocytes [7], tenocytes [8], muscle cells [9], fibroblasts [10], endometrial cells [11], and corneal cells [12].

The importance of the cellular stimulation carried out by PRP is relevant not only to the direct effect on the patient’s tissue after its administration but also in ex vivo processes as an adjuvant to other biological treatments. For example, their use in mesenchymal stem cells for cell therapies could be a breakthrough in this type of treatment. Several studies showed that culturing cells from different niches using PRP improved their bioactivity without altering their unique properties [13,14].

These actions of PRP are ultimately triggered by growth factors and other released cytokines that act through the membrane receptors of the target cells. These PRP effector molecules can be divided into those mainly secreted by platelets, such as transforming growth factor beta (TGF-β), platelet-derived growth factor (PDGF), and vascular endothelial growth factor (VEGF), and those circulating in plasma, such as insulin-like growth factor-1 (IGF-1) or hepatocyte growth factor (HGF). All of these factors are involved in the stimulation of cellular processes such as proliferation, mitogenesis, angiogenesis, migration, and differentiation [15]. 

Since these molecules are the primary effectors of PRP on cellular activity, it is essential to understand if the cytokines as well as the different molecular balances, such as between platelet and plasmatic growth factors, could influence the efficacy of PRP. For this, it is crucial to know the composition of this biological product in order to optimize the results of this treatment as far as possible. 

Thus, the present work was based on the hypothesis that the concentrations of biomolecules present in PRP influence the activation and modulation of cellular processes. Bearing this in mind, the aim of this study was to analyze the influence of particular platelet factors on cell proliferation comparing platelet-rich plasma (PRP) and platelet-poor plasma (PPP), as well as the relationship between cell proliferation and the levels of certain growth factors in PRP of different individuals.

## 2. Results

### 2.1. Platelet-Rich Plasma and Platelet-Poor Plasma Characterization

The mean PRP platelet concentration was 446 ± 142 × 10^3^ platelets/µL, reaching a concentration factor of 2.69, with no leukocytes or erythrocytes. In accordance with the UCS (universal coding system) and minimum reporting requirements for PRP studies, the PRP type was 14-00-11 [16] and the characteristics of the PRP are reported in Table 1. For PPP, no platelets, erythrocytes, or white blood cells were counted in the analysis.

### 2.2. Comparison between Platelet-Rich Plasma and Platelet-Poor Plasma

**Figure 1:**
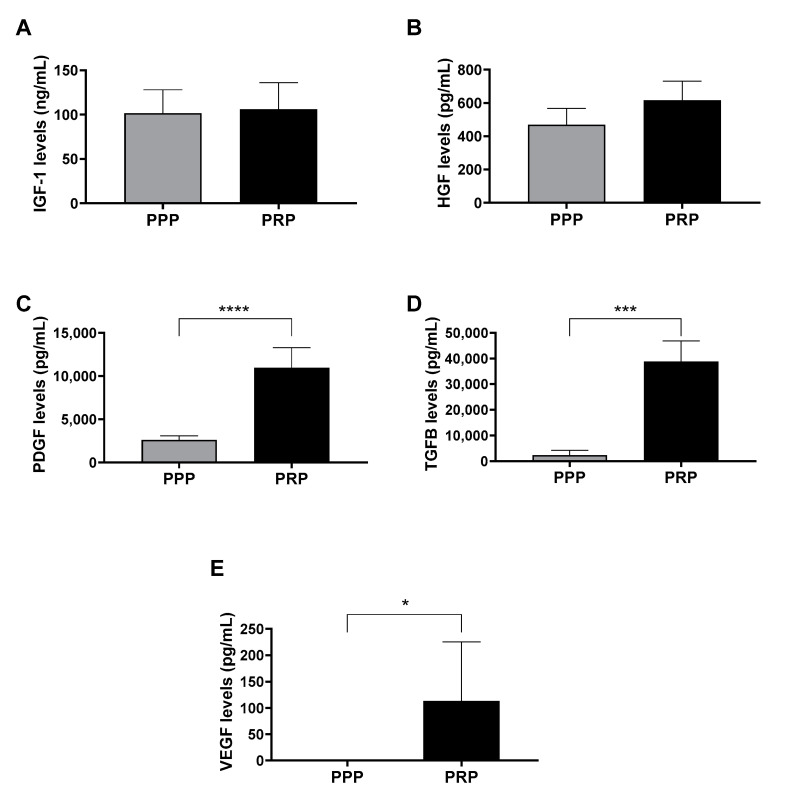
Growth factor levels in lysates after the activation of platelet-poor plasma (PPP) and platelet-rich plasma (PRP). (**A**) Insulin-like growth factor 1 (IGF-1), (**B**) hepatocyte growth factor (HGF), (C) platelet-derived growth factor (PDGF), (**D**) transforming growth factor beta (TGF-β), and (**E**) vascular endothelial growth factor (VEGF) protein levels were measured in duplicate by enzyme-linked immunosorbent assay (ELISA) in the PRP and PPP lysates. Any statistical significance of the differences between PPP and PRP was determined by *t*-test (* *p* < 0.05; *** *p* < 0.001; **** *p* < 0.0001) (*n* = 5).

As a first approach, in order to compare the content of growth factors of PPP and PRP of 5 donors, ELISA assays were performed. The results showed differences in platelet growth factors levels (PDGF, TGF-β, and VEGF), which was consistent with the difference in the presence of platelets between the two lysates compared. However, there were no differences in plasmatic growth factor (IGF-1 and HGF) levels (Figure 1).

**Figure 2:**
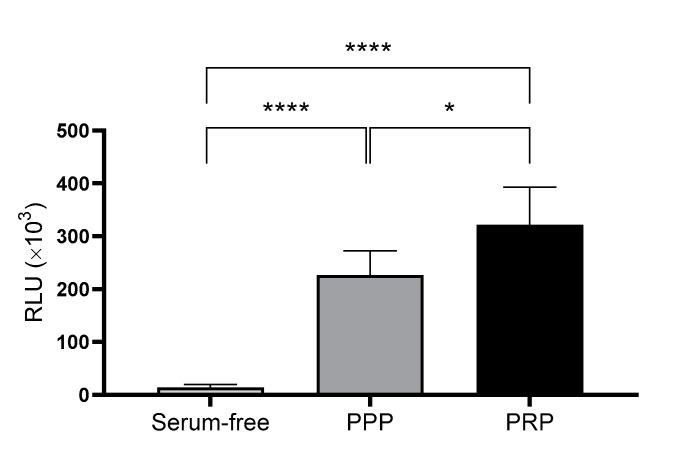
Viability of dermal fibroblasts cultured using a medium supplemented with either platelet-poor plasma (PPP) or platelet-rich plasma (PRP). Viability was measured by luminescence as relative light unit (RLU) after 96 h incubation under each condition. Serum-free medium was used as the negative control. The statistical significance of the differences in viability levels between serum-free control and PPP and PRP supplementation was determined by ANOVA test (* *p* < 0.05; **** *p* < 0.0001) (*n* = 5).

In addition to their content, the ability of the two types of lysates to promote cell proliferation was compared in cultured cells. To this end, cell viability assays were performed comparing the two plasmas, where RLU levels were directly associated with cellular viability. The results indicated that PRP promoted significantly higher viability than PPP at 96 h. Moreover, both conditions were run in parallel with a negative control that contained no serum supplementation (Figure 2).

### 2.3. Analysis of the Effect of Platelet-Rich Plasma Growth Factors on Cell Proliferation

**Figure 3:**
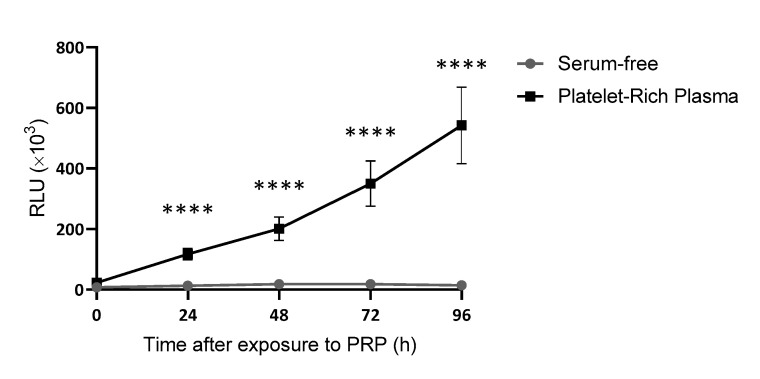
Growth curves of dermal fibroblasts cultured with PRP or serum-free. Cell viability was measured every 24 h for a total of 96 h. The statistical significance of the differences in proliferative capacity between the two conditions was determined by *t*-test at each time-point (**** *p* < 0.0001) (*n* = 30).

**Figure 4:**
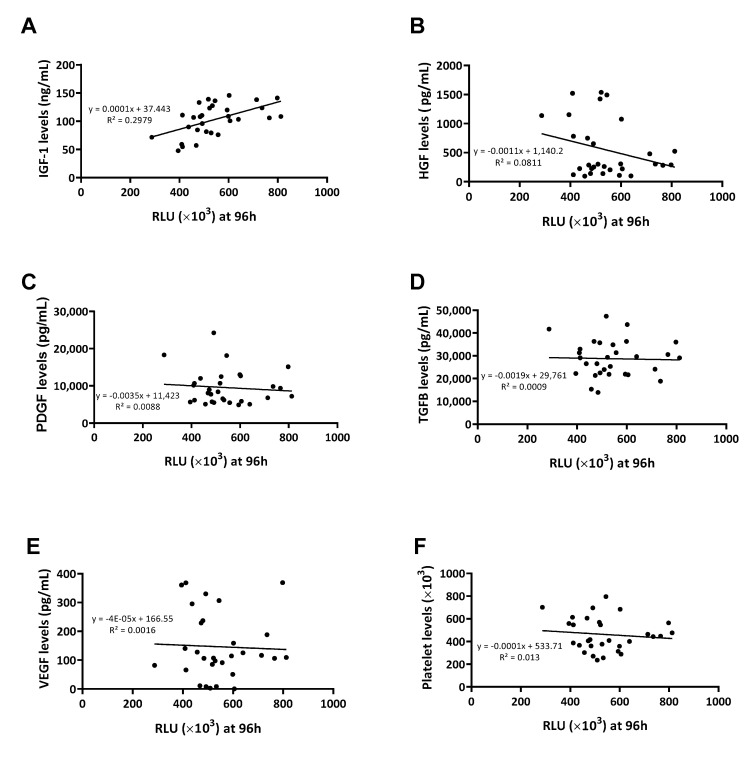
Correlations between growth factor levels and cellular viability. Cell viability was measured at 96 h and the RLU was correlated with IGF-1 (**A**), HGF (**B**), PDGF (**C**), TGF-β (**D**), VEGF (**E**), and platelet (**F**) levels measured by ELISA (*n* = 30).

Considering this first approach, a new batch of 30 samples was employed to establish whether correlations might exist between levels of growth factors within the plasma and their ability to promote cell proliferation. This was only conducted with PRP samples, as PPP samples contained hardly any platelet growth factors. After measuring the effect of PRP on cell proliferation in real time at different time points (Figure 3), an assessment was made of the correlation between the levels of each growth factor or platelet content and the ability of PRP to promote cell proliferation (Figure 4). The only growth factors that showed any tendency to potentially influence the cellular response were plasmatic factors (Figure 4A,B) and not platelet factors (Figure 4C–E) nor platelet levels (Figure 4F).

**Figure 5:**
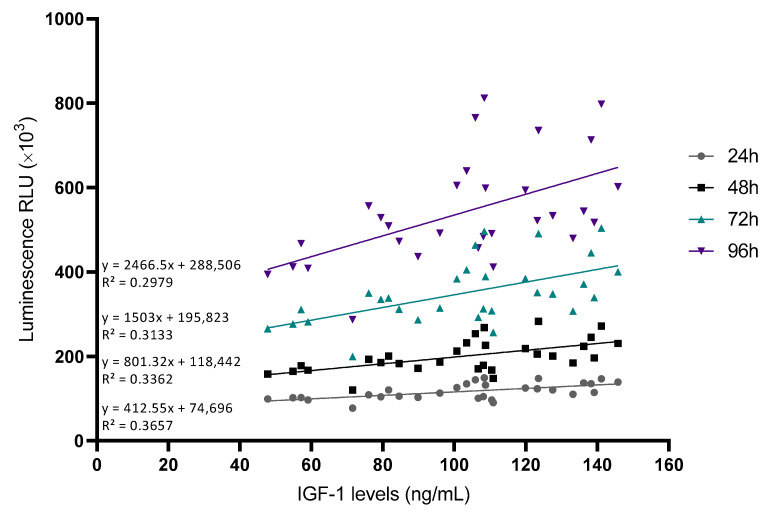
Correlations between IGF-1 levels and cellular viability at different time points. Cell viability was measured at different time points and the RLU was correlated with IGF-1 levels measured by ELISA (*n* = 30).

To understand the statistical relationship between the two variables, Pearson correlations were performed (Table 2). The growth factor that positively influenced the plasma’s ability to promote cell proliferation was found to be IGF-1 at any measured time point (Figure 5).

At the same time, platelet counts were correlated with growth factor levels to see if there was a relationship between them. The results indicated that all of the growth factors, except IGF-1, were affected by the level of platelets in the PRP samples (Table 3).

The multivariate linear regression model indicated that IGF-1 (*p* = 0.001) significantly favored cell proliferation at 96 h. In contrast, HGF had a significantly negative effect on cell proliferation (*p* = 0.047) (Table 4).

## 3. Discussion

Modulation of cellular activity is one of the mechanisms that explain the therapeutic action of PRP, thus stimulating processes such as cell proliferation. The main findings of this work showed that cell proliferation was higher in cells incubated with lysates derived from PRP compared to those cultured with lysates derived from PPP, suggesting that platelet-derived growth factors are essential to enhance this bioactivity. However, and surprisingly, when analyzing the contribution of different growth factors present in PRP samples of similar platelet concentration on cell proliferation, the only growth factor that correlated directly with cellular activity was IGF-1, which was not dependent on platelet number.

It is an undeniable fact that the growth factors released by platelets have a biological action that favors cellular processes. This was demonstrated not only in the present work comparing lysates with and without platelet content, but also in several previous works in which the PRP concentration in the culture medium was shown to influence cell proliferation. Hahn et al. observed a dose-dependent increase in chondrocyte proliferation in vitro by culturing them with different concentrations of platelet concentrate [17]. This dose-dependent effect of PRP has also been reported in other cell populations, such as fibroblasts [18] and mesenchymal cells [19]. Indeed, it appears that the greater effect achieved at higher doses of PRP is ultimately conditioned by the presence of platelet growth factors such as PDGF or TGF-β, the proliferation-stimulating capacities of which are well known [15] and they are correlated with platelet concentration [20]. Ogino et al. showed that in vitro proliferation of osteoblast-like cells was dose-dependent for PDGF and TGF-β [21]. Furthermore, in another study, the proliferative effect of PRP was decreased by the addition of PDGF-neutralizing antibodies [19]. On a clinical level, and although further research is still needed in this field, it seems that a direct correlation has been found between doses of PDGF and TGF-β and improvement in patients with knee osteoarthritis after PRP treatment [22]. Therefore, it would seem that the best approach to improve this treatment would be to use PRP with the highest platelet concentrations. However, the studies mentioned above analyzed PRP with very different platelet concentrations and therefore very different levels of platelet molecules. This could accentuate the dose-dependent effect of platelets in PRP, masking the possible effects of other biomolecules.

In our work, however, all samples were of the same type of PRP and there were no major differences between their platelet concentrations. This was evidenced by the fact that cell proliferation did not correlate with either platelet number or platelet growth factors, although these two variables did correlate with each other. Surprisingly, the only factor that correlated with cell proliferation individually was IGF-1, being the only molecule that did not depend on platelet concentration. This finding was in line with numerous studies in which IGF-1 stimulated cell proliferation in different cell types, such as retinal cells [23], neurons [24], chondrocytes [25], or tenocytes [26]. Therefore, although it seems reasonable to consider the importance of IGF-1 in PRP, no previous studies have demonstrated its dose-dependent effect on cell proliferation after PRP use in cell culture. Furthermore, when performing multiple linear regression analysis, HGF was another factor that significantly influenced the model, although it’s effect depended on platelet levels, and it is highly present in PPP. In this case, it appears that high concentrations of HGF may decrease cell proliferation, which was also shown in previous studies [27,28]. In particular, Yi et al. observed that overexpression of HGF in cardiac fibroblasts activated the c-Met-Akt-TGIF signaling pathway, resulting in inhibition of TGF-β-activated cell proliferation [27]. This could explain the inverse correlation between HGF levels and cell proliferation. However, according to the results of the present study, the magnitude of the positive effect of IGF-1 outweighed the possible negative effect of HGF, which, on the other hand, is key in other mechanisms of action of PRP, such as the modulation of fibrosis [29] or inflammation [30]. These findings highlight the importance of the composition of PRP in the effectiveness of this biological therapy in terms of its cellular composition (platelets and white blood cells) as well as its molecular composition involving biomolecules that do not depend on the presence of platelets or white blood cells. The importance of plasma content in the action of PRP was highlighted in a study carried out by Van der Bijl et al. in which they compared plasma, platelet concentrate, and the combination of the two on cell proliferation. The results showed that the synergistic effects of plasma and platelet concentrate were much more pronounced than those of the separate formulations, demonstrating that the action of PRP depends on the combined action of all components [31].

The modulation of different PRP molecules is important not only in their role as direct effectors of PRP action but also in their interaction with each other. Excessive disturbance of the balance between the molecules that comprise PRP could negatively affect the efficacy of PRP or even generate unwanted effects. [32]. However, modulation at the molecular level is currently a difficult challenge for which the development of new techniques becomes imperative, such as the use of antibodies that selectively neutralize growth factors [33]. It should be noted that the alteration of plasmatic molecules also depends on certain biological factors of the donor. Several studies showed that PRP from aged individuals had lower levels of IGF-1 compared to that of younger subjects [34,35]. Thus, the use of donor PRP may be a valid option to achieve an optimal molecular composition.

Taking into consideration all of the above, it appears that enhancing the effect of PRP on cell proliferation goes beyond a mere increase in platelet dose. Previous studies demonstrated that although a dose-dependent effect of platelets can be observed, the increase in platelets above certain levels not only fails to enhance cellular activity but even seems to have an inhibitory effect [36,37,38]. Therefore, other platelet-independent molecules should not be forgotten when analyzing and optimizing the efficacy of PRP since maintaining the balance between the different types of molecules seems essential to achieving this goal. In this case, the present results suggest that a decrease in IGF-1 levels in relation to platelet growth factor levels has a negative effect on cellular activity. 

The main limitation of this study was inherent to in vitro studies since the results obtained are not always easy to translate to clinical outcomes. In addition, the only cellular process analyzed was cell proliferation, which is, nonetheless, a well-established method to assess the influence on cellular activity. Further preclinical and clinical studies are needed to do more thoroughly assess the therapeutic potential of this product.

## 4. Materials and Methods

### 4.1. Sample Collection

Blood samples for the preparation of PRP and PPP were obtained from healthy donors who did not have any chronic diseases and were not taking any medication. Furthermore, they did not present any hematological alterations according to their hemograms and the serological analysis.

Two groups of patients were included for this study. A first group of 5 donors was used for comparisons between PRP and PPP. A second group of 30 samples was employed for the analysis of the different variables influencing the effect of PRP on cell proliferation. 

### 4.2. Platelet-Rich Plasma and Platelet-Poor Plasma Preparation

PRP and PPP were obtained from whole blood collected in 10 mL tubes containing 3.8% (*w*/*w*/*v*) sodium citrate. To obtain PRP, the blood was centrifuged at 580× *g* for 8 min at room temperature, and the lower 2 mL of the plasma column was collected without including the erythrocyte or leukocyte layers. Thus, the obtained plasma had a concentration of platelets higher than that of blood and an absence of erythrocytes and leukocytes. To obtain PPP, the blood was centrifuged at 1500× *g* for 15 min at room temperature, achieving a plasma fraction free of platelets and any cellular elements. For growth factor and cell proliferation analyses, both plasmas were activated by the addition of CaCl_2_ (10% *w*/*v*), triggering the release of platelet contents (in the case of PRP) and the formation of fibrin clots. After clot formation, they were squeezed and discarded to obtain the lysates of both preparations [1]. These were kept at −80 °C until further use.

### 4.3. Platelet-Rich Plasma and Platelet-Poor Plasma Characterization

The platelet content of PRP and PPP was measured on the Sysmex XS-1000i hematological analyzer (Sysmex, Kobe, Japan). This measurement verified that PRP was correctly prepared and that it complied with the parameters indicated by the PRP kit manufacturer. This hematological analysis was used to confirm the absence of cells and platelets in PPP.

### 4.4. Enzyme-Linked Immunosorbent Assay (ELISA)

To determine the growth factor content of the lysates derived from PRP or PPP, ELISA assays were performed to measure the levels of the following proteins according to the manufacturer’s instructions: VEGF (DVE00; Bio-techne, Minneapolis, MI, USA), TGF-β (DB100B; Bio-techne), IGF-1 (DG100; Bio-techne), HGF (DHG00B; Bio-techne), and PDGF (DHD00C; Bio-techne). Growth factor levels were measured by absorbance and the corresponding concentrations were calculated by means of calibration curves.

### 4.5. Cell Cultures

Normal human dermal fibroblasts (CC-2511; Lonza, Gampel-Bratsch, Switzerland) were used to measure the bioactivity of PRP or PPP in vitro. The cells were maintained in an incubator at 37 °C and 5% CO_2_ with basal fibroblast growth medium (CC-3131; Lonza) supplemented with insulin, human fibroblast growth factor, and gentamicin-amphotericin sulphate at 0.1% (*v*/*v*) each (CC-4126), according to the manufacturer’s recommendations. In addition, the corresponding PRP lysate was added for each condition at 10% concentration. An unsupplemented medium was used as negative control.

### 4.6. Cell Viability Assay

To assess the biological activity of PRP and PPP, dermal cells were incubated with media supplemented with platelet lysate and real-time monitoring of cell viability was performed over a period of 96 h. Cell viability was measured using the Realtime-Glo MT cell viability assay (G9711; Promega, Madison, WI, USA), which is based on the reducing potential of metabolically active cells in catalyzing the conversion of a synthetic substrate into a luminescent product. Thus, the luminescence measured in relative light units (RLU) by the detector was proportional to the number of viable cells.

### 4.7. Statistical Analysis

Variables were determined by the mean and standard deviation for parametric data, and median and 95% confidence interval (CI) for non-parametric data. Comparisons were performed by Student’s *t*-test for independent or paired parametric data, the Wilcoxon signed-rank test for paired non-parametric data, and the Mann–Whitney U test for independent non-parametric data. Multivariate linear regression was performed to analyze the influence of the different variables taken collectively, calculating coefficients (B and beta), *p* value, and 95% CI. Distribution of the samples was assessed by Shapiro–Wilk’s test. Data were considered statistically significant when *p* < 0.05. Statistical analysis was performed using SPSS 20.0 software (SPSS, Chicago, IL, USA).

## 5. Conclusions

Lysates derived from PRP enhanced the cell proliferation compared to using lysates derived from PPP. Furthermore, levels of platelet growth factors such as PDGF and TGF-β in samples of the same type of PRP had no influence on variations in cellular activity. In contrast, increased IGF-1 levels, which are not dependent on platelet concentration, were directly correlated with enhanced cell proliferation.

## Figures and Tables

**Table 1:** Characteristics of PRP.

PRP Preparation	
Blood volume taken	8 mL
Anticoagulant	Sodium citrate 3.8% (*w/v*)
System	Close
Centrifugation	Once, 580× *g*/8 min
Final PRP volume	2 mL
**PRP Characteristics**	
PRP Type	14-00-11
MPV	10.4 fL (CI: 9.40–11.60)
Red Blood Cells	<0.01 × 10^6^/μL
White Blood Cells	<0.05 × 10^6^/μL
Activation	CaCl_2_ 10% (*w/v*)
**Application Characteristics**	
Dose	10%
Direct/Indirect	Direct
Cell line	Normal human dermal fibroblasts
**Other Remarkable PRP and Study Features**	
The product added to the cell cultures was the platelet lysate obtained after activation of PRP using calcium chloride (10%)	
PRP: platelet-rich plasma; IA: intraarticular; IO: intraosseous; MPV: mean platelet volume	

**Table 2:** Correlations between growth factors and cellular viability levels represented in RLU.

		IGF-1 Levels	HGF Levels	PDGF Levels	TGF-β Levels	VEGF Levels	Platelet Levels
**RLU at 24 h**	Pearson correlation	**0.605**	−0.115	−0.065	0.018	−0.076	−0.057
	Sig. (bilateral)	0.000 ****	0.544	0.732	0.924	0.689	0.766
**RLU at 48 h**	Pearson correlation	**0.58**	−0.177	−0.071	−0.023	−0.049	−0.069
	Sig. (bilateral)	0.001 ***	0.35	0.71	0.903	0.798	0.715
**RLU at 72 h**	Pearson correlation	**0.56**	−0.216	−0.067	−0.015	−0.042	−0.07
	Sig. (bilateral)	0.001 ***	0.253	0.725	0.936	0.826	0.714
**RLU at 96 h**	Pearson correlation	**0.546**	−0.285	−0.094	−0.03	−0.041	−0.114
	Sig. (bilateral)	0.002 **	0.127	0.622	0.873	0.831	0.549

The statistical significance was calculated by Pearson correlation. (** *p* < 0.01; *** *p* < 0.001; **** *p* < 0.0001) (*n* = 30). Heatmap according to Pearson values.

**Table 3:** Correlations between growth factors and platelet levels.

		IGF-1 Levels	HGF Levels	PDGF Levels	TGF-β Levels	VEGF Levels
**Platelet levels**	Pearson correlation	0.02	**0.782**	**0.685**	**0.666**	**0.396**
	Sig. (bilateral)	0.915	0.000 ****	0.000 ****	0.000 ****	0.030 *

The statistical significance was calculated by Pearson correlation. (* *p* < 0.05; **** *p* < 0.0001) (*n* = 30). Heatmap according to Pearson values.

**Table 4:** Multivariate regression analysis for cell proliferation at 96 h.

	B	Beta	*p* Value	95% CI	
**IGF-1**	2745.017	0.607	0.001 ***	1206.009	4284,024
**HGF**	−146.809	−0.564	0.047 *	−291.848	−1.769
**PDGF**	−8.033	−0.297	0.232	−21.558	5.493
**TGF-β**	−0.692	−0.044	0.851	−8.237	6.853
**VEGF**	−225,451	−0.205	0.313	−677.801	226.898
**Platelets**	539.127	0.629	0.092	−94.322	1172.575

B: unstandardized coefficient; Beta: standardized coefficient; CI: confidence interval. (* *p* < 0.05; *** *p* < 0.001) (*n* = 30).

## Data Availability

The data presented in this study are available within the article. Additional inquiries may be directed to the corresponding authors.
